# Antibiotic Prescription Practice and Resistance Patterns of Bacterial Isolates from a Neonatal Intensive Care Unit: A Retrospective Study from Jordan

**DOI:** 10.3390/antibiotics14010105

**Published:** 2025-01-18

**Authors:** Mariam Alameri, Lobna Gharaibeh, Mervat Alsous, Aseel Yaghi, Asma’a Tanash, Saqr Sa’id, Hanan Sartawi

**Affiliations:** 1 Department of Clinical Pharmacy and Pharmacy Practice, Faculty of Pharmacy, Yarmouk University, Irbid 21163, Jordan; mervat.alsous@yu.edu.jo; 2 Biopharmaceutics and Clinical Pharmacy Department, Faculty of Pharmacy, AI-Ahliyya Amman University, Amman 11941, Jordan; l.gharaibeh@ammanu.edu.jo (L.G.); aseelreziqaliyaghi@gmail.com (A.Y.); 3 Clinical Pharmacy Department, Al Basheer Government Hospital, Ministry of Health, Amman 11941, Jordan; asmatanash@gmail.com; 4 Microbiology Department, Al Basheer Government Hospital, Ministry of Health, Amman 11941, Jordan; alsalahatsaqer@gmail.com; 5 Pharmacy and Clinical Pharmacy Directorate, Ministry of Health, Amman 11941, Jordan; hanan.sartawi@moh.gov.jo

**Keywords:** neonatal sepsis, empirical antibiotic, antibiotic resistance, vancomycin, neonatal intensive care unit, Jordan

## Abstract

**Background/Objectives**: Neonatal sepsis is a systemic inflammation in neonates caused by bacteria, viruses, or fungi that can progress into severe conditions. In developing countries, neonatal sepsis is a major cause of mortality and a major public health issue with a high prevalence. This study aims to evaluate the antibiotic prescription practice and resistance patterns of bacterial isolates from the neonatal intensive care unit (NICU) at the largest governmental hospital in Amman, Jordan. **Methods**: This was a retrospective cross-sectional study. The antibiotic prescription practice and resistance patterns of bacterial isolates from the NICU at Al Basheer Government Hospital in Amman, Jordan, were evaluated. The hospital’s microbiology lab database and medical records were the sources of the retrospective data collection. **Results**: A total of 266 neonates treated with antibiotics were assessed. The findings showed that most neonates had late-onset sepsis (LOS) (65.4%). The penicillin group of antibiotics (ampicillin) was the most highly prescribed first empiric antibiotic for LOS and early-onset sepsis (EOS) (61.7%). Aminoglycosides (60.9%) were the most prescribed antibiotics as a second empiric treatment for EOS and LOS. The culture results showed that resistance to antibiotics was as follows: 15.4% of the culture samples were resistant to penicillin (*Micrococcus* and *Viridans streptococci*), 13.9% were resistant to cefotaxime (*Klebsiella pneumoniae* and *Viridans streptococci*), 13.2% were resistant to cefoxitin (*Klebsiella pneumoniae* and *Staphylococcus epidermidis*), and 12.4% were resistant to oxacillin (*Klebsiella pneumoniae* and *Staphylococcus epidermidis*). **Conclusions**: This retrospective study sheds light on the antibiotic prescription practice and resistance patterns of bacterial isolates from newborns with sepsis. The results highlight the high rates of antibiotic resistance. These findings underline the urgent need for improved antibiotic stewardship and infection control strategies to prevent resistance from spreading further.

## 1. Introduction

Neonatal sepsis is a systemic inflammation in neonates caused by bacteria, viruses, or fungi that can progress into severe conditions [1]. Rudd et al. examined the global burden of sepsis and its incidence patterns from 1990 to 2017. Their study revealed a decrease in sepsis incidence by 37.0% from 1990 to 2017 and a drop in mortality by 52.8% [2]. Sepsis incidence and mortality are inconsistent worldwide and vary considerably among regions [3]. In developing countries, neonatal sepsis is a major cause of mortality and a major public health issue with a prevalence as high as 29.92% [4].

Neonatal sepsis is categorized into two groups depending on the time of onset. Early-onset neonatal sepsis is a confirmed infection in the blood or cerebrospinal fluid within the first 72 h of life, and late-onset if it occurs between 3 and 28 days [5]. Some experts provide a cut-off time of 7 days to define the early and late onset of neonatal sepsis [6,7]. EOS is an infection that occurs before and/or during delivery (from the female genitourinary system), whereas late-onset most probably occurs through the surrounding environment after delivery such as hospitals or caregivers [8,9].

EOS involves several types of bacteria such as *Streptococcus agalactiae*, *Escherichia coli*, *Staphylococcus aureus*, *Enterococcus*, and *Streptococcus pneumonia*. However, LOS includes coagulase-negative staphylococci, *Klebsiella pneumoniae*, and *Acinetobacter baumannii* [10].

A recent review by Glaser et al. revealed that the most prevalent pathogens in EOS are Group B *Streptococcus* and *Escherichia coli*, and coagulase-negative *staphylococci* for LOS [11].

However, these epidemiological data may differ based on the setting. Dong et al., in a review of neonatal sepsis in China, reported that the prevalence of Group B *Streptococcus* in EOS differs from that in developed countries [12].

Similarly, Russel et al.’s review showed distinct differences in the etiology between low-, middle-, and high-income countries [13].

Neonatal sepsis is a severe disease with detrimental consequences; therefore, empirical therapy with broad-spectrum antibiotics is necessary for neonates with risk factors and/or suspected sepsis [14]. However, the use of inappropriate broad-spectrum antibiotics and for a longer-than-needed duration in neonatal intensive care units leads to the emergence of multidrug-resistant micro-organisms, which limits the number of effective antibiotics [15]. Antibiotic susceptibility patterns, which guide the antibiotic profile used, differ from one institution to another. Wang et al. reported that most isolated coagulase-negative *staphylococci* specimens were resistant to β-lactam drugs [16]. Pokhrel et al.’s study showed that coagulase-negative *staphylococci* were resistant to oxacillin, cefotaxime, and meropenem, but susceptible to vancomycin and linezolid [17]. Tan et al. evaluated antibiotic resistance in neonatal invasive bacterial infections in China from 2012 to 2019. Their study reported that all *Staphylococcus aureus* specimens were not susceptible to ampicillin and penicillin and half of these cultures were resistant to methicillin. Additionally, all Group B *Streptococcus* were susceptible to penicillin and ampicillin, but most frequently resistant to erythromycin and clindamycin [18]. Based on previous evidence, medical institutions should assess antibiotic resistance patterns regularly to identify adequate and effective antibiotics.

This study aims to evaluate the antibiotic prescription practice and resistance patterns of bacterial isolates from the neonatal intensive care unit at AlBasheer Hospital, the largest governmental hospital and neonatal intensive care unit, in Amman, Jordan.

## 2. Methodology

This was a retrospective cross-sectional study. Antibiotic resistance patterns and microbiological features in the NICU at Al Basheer Government Hospital in Amman, Jordan, were evaluated in this study. November 2022 to May 2024 was the study’s period. A reference number (17371/IRB) for the IRB approval was obtained from the Jordanian Ministry of Health.

### 2.1. Study Sittings

One of the biggest public hospitals in Amman, Jordan, Al Basheer Hospital, handles a sizable number of neonatal patients, making it the perfect location for the study’s assessment of antibiotic resistance in this group. The NICU, which admits newborns in need of acute care owing to a variety of infections, preterm, and other issues, was the specific focus of the study. This NICU usually has approximately 3–4 surgery patients per month. Most patients are ventilated with mechanical ventilation or with a nasal cannula. Moreover, all the neonates with very low or low birth weight (<1.5 kg) in the very preterm category were on total parenteral nutrition (TPN), had intravenous catheters, and were ventilated.

### 2.2. Study Participants and Data Collection

The hospital’s microbiology lab database and medical records were the sources of the retrospective data collection. The data that was obtained was as follows: Neonate demographic information, including age, gender, gestational age, and birth weight. Clinical information, such as the diagnosis, length of NICU stay, and underlying health issues. Microbiological information, such as the kind of bacteria that were isolated from blood. Patterns of antibiotic susceptibility as established by the hospital’s laboratory. Treatments with antibiotics, classes, and specific agents. The microbiological method used in this hospital was the conventional culture method for microbial isolation. Strict criteria were used to differentiate between true pathogens and contaminants according to the standard operating procedures (SOPs) and Clinical & Laboratory Standards Institute (CLSI) guidelines. The guidelines stress the importance of sterile collection, proper preparation, and adequate blood volume for reliable results. Moreover, the guideline recommends collecting 2, or preferably 3, blood culture sets for each septic episode. A contaminant will usually be present in only one bottle of a set of blood culture bottles, in contrast to a true bloodstream infection, in which multiple blood culture bottles/sets will be positive.

### 2.3. Statistical Analyses

Statistical software, such as SPSS version 25, was used to evaluate the data once they were entered into a secure database. The microbiological data, antibiotic prescription practice, antibiotic resistance patterns, clinical aspects, and demographics were all summarized using descriptive statistics. Categorical variables were expressed as frequencies and percentages, and continuous variables were provided as means (±standard deviation). A *p*-value of ≤0.05 was considered statistically significant.

### 2.4. Ethical Approval

The Jordanian Ministry of Health’s Institutional Review Board (IRB) gave its approval to this study (Reference number: 17371/IRB). Since the study was retrospective, the patients’ or their guardians’ informed consent was unnecessary. To maintain confidentiality, all the data were anonymized, and only the research team had access to them.

## 3. Results

### 3.1. Characteristics of the Participants

The prescription practices related to antibiotics and patterns of antibiotic resistance of bacterial isolates obtained from neonates with sepsis were assessed over eighteen months. During this period, 266 neonates who were treated with antibiotics were assessed. The majority (97.0%) of the neonates were admitted for the first time to the hospital. The mean gestational age was 35 days. Only 12% of the neonates were born preterm with very low birth weight. About half of the patients were male (56.8%) and delivered by cesarean section (59.4%). Signs of infection included chest retraction (19.9%), high levels of CRP (19.1%), and tachypnea (13.2%). More than one-third of the neonates were diagnosed with respiratory distress syndrome (RDS). Most neonates had LOS (65.4%). The late onset of sepsis was higher in the male neonates (*n* = 107), those born preterm (*n* = 29), and those with low birth weight (*n* = 26) [*p*-value = 0.032, 0.003, and 0.031, respectively] compared to the females and those born on term and with normal gestational weight. The mortality rate among the neonates in this study was (1.88%). Characteristics of the neonatal patients are summarized in Table 1.

### 3.2. Characteristics of Antibiotic Prescription for the Treatment of Neonatal Sepsis

In this study, the range of empiric antibiotics used consecutively was between 0 and 6. About 70% of the neonates were treated with two antibiotics, and only 0.8% were treated with six antibiotics. None of the neonates received antibiotics to treat intra-amniotic infection, while 1.5% percent only of their mothers received prenatal antibiotics. The physician decided to change the antibiotic treatment after the results of the specimen culture in 45.1% of the neonates, where the number of targeted antibiotics ranges between 1 and 4 antibiotics, as seen in Table 2.

The mean number of empirical antibiotics used in LOS was higher than in EOS (2.32 vs. 2.0, *p*-value 0.013).

### 3.3. Types of Bacterial Isolates from Neonatal Sepsis Patients in Jordan

The most common types of Gram-positive bacterial isolates were *Micrococcus luteus* (37.5%), *Staphylococcus epidermidis* (36.2%), and *Streptococcus viridans* (6.9%). On the other hand, the most common types of Gram-negative bacterial isolates were *Klebsiella pneumonia* (41.2%), *Acinetobacter baumanni* (17.6%), and *Escherichia coli* (14.7%). For Gram-positive bacteria in late-onset sepsis, the most common cause was *Staphylococcus epidermidis* (25.9%) followed by Micrococcus luteus (22.8%). The results are shown in Table 3.

### 3.4. Practice of Antibiotic Prescription for Neonatal Sepsis Patients in Jordan

The penicillin group of antibiotics (ampicillin) was the most highly prescribed first empiric antibiotic for neonates (61.7%), followed by dehydropeptidase/carbapenems such as meropenem (11.3%) and glycopeptides (10.9%). Aminoglycosides (60.9%) were the most prescribed antibiotics as a second empiric treatment. On the other hand, polymixins and nitroimidazole were the least prescribed for the treatment of neonatal infections. The results of antibiotic prescription practices for neonatal sepsis are described in Table 4.

The results of the specimen culture were available for 232 of the participants, and treatment was amended according to the culture results. Vancomycin was prescribed as a replacement for the previous antibiotic (34.2%), followed by aminoglycosides (28.3%). If the physician decided to make another change in the treatment, meropenem was most prescribed (32.5%). Only third-generation cephalosporins and meropenem were an option in all numbers of changed antibiotics, as shown in Table 5.

### 3.5. Resistance Patterns to Antibiotics Used for Neonatal Sepsis Patients in Jordan

The culture results showed that resistance to antibiotics was as follows: 15.4% of the culture samples were resistant to penicillin (*Micrococcus* and *Viridans streptococci*), 13.9% were resistant to cefotaxime (*K. pneumonia* and *Viridans streptococci*), 13.2% were resistant to cefoxitin (*K. pneumonia* and *Staph. Epidermis*), and 12.4% were resistant to oxacillin (*K. pneumonia* and *Staph. Epidermis*). Details are shown in Table 6.

## 4. Discussion

The results of this study shed light on the antibiotic prescription practice and resistance patterns of bacterial isolates from newborns with sepsis hospitalized in a Jordanian NICU. During the study period, neonates receiving antibiotic treatment for sepsis were assessed, indicating many noteworthy clinical features, prescription patterns, and microbiological resistance patterns.

Regarding the neonates’ demographics, it appears that term infants with a mean gestational age of 35 days made up the majority of the cases. On the other hand, most neonates in the current study had late-onset sepsis, especially those with low birth weight and preterm. This result is consistent with earlier research, which found that low birth weight and preterm delivery were major risk factors for neonatal sepsis because of the immune system’s immaturity and extended NICU stays, which could expose patients to more nosocomial infections [19,20]. The gender disparity, where the male newborns had a higher risk of late-onset sepsis, is also in line with previous research suggesting that immunological or hormonal disparities may make male neonates more susceptible to infections [21].

The study’s antibiotic use patterns show that penicillin, particularly ampicillin, is the most common first-line empiric therapy for neonatal sepsis. This preference may be due to ampicillin’s documented efficiency in targeting the most frequent bacteria in EOS, including Group B *Streptococcus* and *Escherichia coli* [22]. The usage of aminoglycosides as a second-line treatment is most likely due to their synergistic effects when taken with other antibiotics, notably in the treatment of Gram-negative infections [23]. According to Hodoșan et al., the combination of ampicillin and gentamicin has been the most effective empirical treatment for sepsis for many years due to its etiological variability [24].

The current study findings show that in about 45% of the cases, the results of the culture were used to modify the empirical antibiotic selection. This is a sign of good clinical practice since it shows that antibiotic resistance may be fought by making adjustments based on microbial sensitivity [25]. Findings, however, indicate that managing sepsis in older neonates is more complex, frequently requiring broader-spectrum antibiotic coverage initially due to increased exposure to nosocomial pathogens. Specifically, LOS was found to be associated with a higher mean number of empiric antibiotics compared to EOS (2.32 vs. 2.0, *p* = 0.013). These findings are consistent with the reported literature. A recent narrative review about antimicrobial resistance among neonates stated that long-lasting hospitalized neonates are more likely to become infected with multidrug-resistant micro-organisms [26].

According to the microbiological reports in this study, *Klebsiella pneumoniae*, *Acinetobacter baumannii*, and *Escherichia coli* dominated the Gram-negative isolates, whereas *Micrococcus luteus* and *Staphylococcus epidermidis* were the most common Gram-positive isolates. Given that *Acinetobacter baumannii* and *Klebsiella pneumoniae* are known to be multidrug-resistant and frequently linked to infections acquired in hospitals [27,28], the high incidence of these bacteria is especially worrying. In addition to being a common contaminant, the presence of coagulase-negative staphylococci like *Staphylococcus epidermidis* may also point to an over-reliance on invasive medical equipment like central venous catheters in this population, which increases the risk of bloodstream infections [29].

The test results for antibiotic susceptibility showed a concerning trend of resistance caused by *Micrococcus*, *Viridans streptococci*, *Klebsiella pneumoniae*, and *Staphylococcus epidermidis*. Resistance rates to commonly used antibiotics—penicillin, cefotaxime, cefoxitin, and oxacillin—were found to be substantial, ranging from 12.4% to 15.4%. These findings align with the worldwide rising antimicrobial resistance (AMR) patterns among different bacterial species [30]. This shows that resistant bacteria in the NICU may have emerged as a result of the overuse or improper usage of these antibiotics [31]. According to a systematic literature review, the findings highlight the fact that the vast majority of infections exhibit strong resistance to widely used antibiotics [32].

According to a recent systematic review, 28 studies revealed that *E. coli* showed high levels of resistance to ampicillin, cephradine, penicillin, and amoxicillin. Thirteen studies revealed that *Klebsiella* spp. had significant resistance to second- and third-generation antibiotics, such as cefaclor and cefotaxime. Fifteen studies indicated high resistance to nearly all the tested antibiotics in *Acinetobacter* spp. [33]. The necessity for careful stewardship in antibiotic prescribing methods to prevent the development of further resistance is highlighted by the comparatively high resistance to gentamicin and trimethoprim/sulphamethoxazole [34]. A recent review about early-onset neonatal sepsis in low- and middle-income countries concluded that over the past ten years, there has been an alarming rise in antimicrobial resistance [35]. As multidrug-resistant organisms can complicate treatment and increase infant mortality, the study’s results highlight the importance of the ongoing observation of antibiotic resistance patterns in NICUs [36]. While important in life-threatening cases such as sepsis, the relatively high incidence of empiric antibiotic treatment prior to culture results should be counterbalanced with timely de-escalation based on susceptibility profiles to minimize the risk of resistance development [37].

A number of actions could be suggested in order to counteract the growing trend of antibiotic resistance: Improved Antibiotic Stewardship Programs: It is imperative to put strong restrictions on the use of antibiotics into place, particularly when it comes to the empirical use of broad-spectrum antibiotics [38,39,40]. Regular audits and feedback on prescribing procedures could help achieve this [39]. Targeted Therapy: By using fast diagnostic tests more frequently, antibiotic therapy can be more quickly tailored, avoiding needless exposure to a wide range of antibiotics [41,42,43]. Moreover, to combat the growing threat of resistance in NICU populations, research into novel antimicrobials and alternative treatments is necessary, especially in light of the growing resistance to important antibiotics like vancomycin [44].

The study’s retrospective methodology has drawbacks because it may result in biases and missing data. The results may not have been as applicable to other NICUs in Jordan or throughout the world because the investigation was restricted to infants admitted to a single hospital. This approach offers a well-defined plan for assessing antibiotic resistance patterns and prescription practice in a susceptible newborn population, highlighting the necessity of longer-term surveillance to obtain more thorough data on antibiotic resistance patterns.

## 5. Conclusions

This retrospective study sheds light on the antibiotic prescription practice and resistance patterns of bacterial isolates from newborns with sepsis in a Jordanian NICU. The results highlight high rates of antibiotic resistance. These findings underline the urgent need for improved antibiotic stewardship and infection control strategies to prevent resistance from spreading further. Despite being therapeutically justifiable, the intense reliance on empirical antibiotic treatment may be a contributing factor to the escalating issue of AMR.

The use of intrapartum antibiotic prophylaxis for high-risk deliveries (such as those with prolonged labor or the preterm rupture of membranes) should be encouraged in Jordan. In addition, we should increase pregnant women’s knowledge of the value of prenatal care, delivery planning, and receiving medical help as soon as possible. Knowing the risks of sepsis and other pregnancy-related issues can help perform vaginal swab screening, encourage early hospital visits, and decrease the need for emergency hospitalizations in regions with limited access to health care. Moreover, poor countries like Jordan may obtain funds and resources for improving newborn care by working with international health organizations like the United Nations Children’s Fund (UNICEF) and the World Health Organization (WHO).

## Figures and Tables

**Table 1:** Study sample characteristics (*n* = 266).

Factor	(Mean ± SD)	
**Gestational age (days)**	35.10 ± 2.90	
Hospital stay (days)	18.46 ± 23.79	
Birth weight (mg)	2424.20 ± 845.42	
**Factor**	**Category**	***n* (%)**
**Gender**	Male	151 (56.8)
	Female	115 (43.2)
**Previous admission**	Yes	8 (3.0)
	No	258 (97.0)
**Signs of infection**	Bradycardia	7 (2.6)
	Abdominal distension	19 (7.1)
	Vomiting	25 (9.3)
	Chest retraction	53 (19.9)
	CRP	51 (19.1)
	Grunting	21 (7.9)
	Tachypnea	35 (13.2)
	Fever	12 (4.5)
	Poor feeding	16 (6.0)
	Hypoactivity	6 (2.3)
	Mottled skin	20 (7.5)
	None	73 (27.4)
**Diagnosis on admission**	Transient tachypnea of the newborn (TTN)	33 (12.4)
	Respiratory distress syndrome	106 (39.8)
	Neonatal jaundice (NNT)	32 (12.0)
	Cyanosis	24 (9.0)
	Intrauterine growth restriction (IUGR)	15 (5.6)
	Vomiting	11 (4.1)
**Birth weight category**	Very low	33 (12.4)
	Low	93 (40.0)
	Normal	140 (52.6)
**Gestational age category**	Very preterm	33 (12.4)
	Moderate to late preterm	96 (36.1)
	Term	137 (51.5)
**Type of delivery**	Normal delivery	102 (38.3)
	Cesarean section	158 (59.4)
	Unknown	6 (2.3)
**Onset of sepsis**	Early onset	92 (34.6)
	Late onset	174 (65.4)
**Early-onset sepsis according to gestational age**	Very preterm	4 (1.5)
	Moderate to late preterm	43 (16.2)
	Term	45 (16.9)
**Late-onset sepsis according to gestational age**	Very preterm	29 (10.9)
	Moderate to late preterm	53 (19.9)
	Term	92 (34.6)
**Patient’s prognosis**	Discharged	260 (97.7)
	Died	5 (1.88)
	Transferred	1 (0.38)

**Table 2:** Characteristics of antibiotic prescription for the treatment of neonatal sepsis.

Factor	Category	*n* (%)
**Prenatal Antibiotic Use**	**No**	**262 (98.5)**
	**Yes**	**4 (1.5)**
**Intrapartum antibiotic use**	No	266 (100)
**Number of empirical antibiotics used**	0	13 (4.9)
	1	11 (4.1)
	2	184 (69.2)
	3	26 (9.8)
	4	29 (10.9)
	5	1 (0.4)
	6	2 (0.8)
**Antibiotics changed after culture**	Yes	120 (45.1)
	No	134 (50.4)
**Number of targeted antibiotics used when changing antibiotics**	1	37 (30.8)
	2	52 (43.3)
	3	22 (18.3)
	4	9 (7.5)
**Antibiotics prescribed in early-onset sepsis**	Penicillin	83 (31.2)
	Dehydropeptidase inhibitor/carbapenem	5 (1.9)
	Glycopeptide	4 (1.5)
	Aminoglycosides	81 (30.5)
	3rd-generation cephalosporin	5 (1.9)
**Antibiotics prescribed in late-onset sepsis**	Penicillin	84 (31.6)
	Dehydropeptidase inhibitor/carbapenem	61 (22.9)
	Glycopeptide	50 (18.8)
	Aminoglycosides	101 (38.0)
	3rd-generation cephalosporin	14 (5.3)

**Table 3:** Type of bacterial infection, Gram-positive bacteria, and Gram-negative bacteria.

A. Type of bacterial infection, Gram-positive bacteria, (*n* = 232).			
**Type of Bacteria**	**Early-Onset Sepsis** ***n* (%)**	**Late-Onset Sepsis** ***n* (%)**	***n* (%)**
*Micrococcus luteus*	34 (14.7)	53 (22.8)	87 (37.5)
*Staphylococcus epidermidis*	24 (10.3)	60 (25.9)	84 (36.2)
*Streptococcus viridans*	7 (3.0)	9 (3.9)	16 (6.9)
*MRSA*	3 (1.3)	11 (4.7)	14 (6.0)
*Diphtheroids (* *Corynebacterium diphtheriae)*	5 (2.2)	1 (0.4)	6 (2.6)
*Enterococcus faecalis*	3 (1.3)	1 (0.4)	4 (1.7)
*Bacillus cereus*	3 (1.3)	0 (0)	3 (1.3)
*Staphylococcus hominis*	1 (0.4)	2 (0.9)	3 (1.3)
*Staphylococcus haemolyticus*	1 (0.4)	1 (0.4)	2 (0.9)
*Staphylococcus aureus*	0 (0)	2 (0.9)	2 (0.9)
*Streptococcus pneumoniae*	1 (0.4)	1 (0.4)	2 (0.9)
*Streptococcus agalactiae*	1 (0.4)	0 (0)	1 (0.4)
B. Type of bacterial infection, Gram-negative bacteria, (*n* = 34)			
**Type of Bacteria**	**Early-Onset Sepsis** ***n* (%)**	**Late-Onset Sepsis** ***n* (%)**	***n* (%)**
*Klebsiella pneumoniae*	2 (5.8)	12 (35.3)	14 (41.2)
*Acinetobacter baumannii*	1 (2.9)	5 (14.7)	6 (17.6)
*Escherichia coli*	1 (2.9)	4 (11.8)	5 (14.7)
*Enterobacter cloacae*	3 (8.8)	0 (0)	3 (8.8)
*Klebsiella oxytoca*	1 (2.9)	0 (0)	1 (2.9)
*Acinetobacter lwoffii*	1 (2.9)	0 (0)	1 (2.9)
*Sphingomonas paucimobilis*	1 (2.9)	0 (0)	1 (2.9)
*Citrobacter koseri*	0 (0)	1 (2.9)	1 (2.9)
*Neisseria sicca*	1 (2.9)	0 (0)	1 (2.9)
*Pseudomonas aeruginosa*	1 (2.9)	0 (0)	1 (2.9)

**Table 4:** The group and name of antibiotics used.

A. Group of antibiotics used (*n* = 266).						
**Antibiotic Group**	**Empiric 1** ***n* (%)**	**Empiric 2** ***n* (%)**	**Empiric 3** ***n* (%)**	**Empiric 4** ***n* (%)**	**Empiric 5** ***n* (%)**	**Empiric 6** ***n* (%)**
**Penicillin**	164 (61.7)	3 (1.1)	0 (0)	0 (0)	0 (0)	0 (0)
**Penicillin–beta lactamase**	1 (0.4)	0 (0)	0 (0)	2 (0.8)	0 (0)	0 (0)
**Dehydropeptidase/Carbapenem**	30 (11.3)	36 (13.5)	22(8.3)	12 (4.5)	2 (0.8)	2 (0.8)
**Glycopeptide**	29 (10.9)	25 (9.4)	10 (3.8)	7 (2.6)	2 (0.8)	0 (0)
**Aminoglycosides**	20 (7.5)	162 (60.9)	11 (4.1)	6 (2.3)	0 (0)	0 (0)
**3rd-generation Cephalosporin**	3 (1.1)	16 (6.0)	4 (1.5)	1 (0.4)	0 (0)	0 (0)
**Nitroimidazole**	2 (0.8)	0 (0)	10 (3.8)	2 (0.8)	0 (0)	0 (0)
**Polymyxins**	0 (0)	0 (0)	0 (0)	1 (0.4)	0 (0)	0 (0)
B. Name of antibiotics used (*n* = 266).						
**Antibiotic**	**Empiric 1** ***n* (%)**	**Empiric 2** ***n* (%)**	**Empiric 3** ***n* (%)**	**Empiric 4** ***n* (%)**	**Empiric 5** ***n* (%)**	**Empiric 6** ***n* (%)**
**Ampicillin**	164 (61.7)	3 (1.1)	0 (0)	0 (0)	0 (0)	0 (0)
**Amikacin**	19 (7.1)	32 (12.0)	10 (3.8)	6 (2.3)	0 (0)	0 (0)
**Meropenem**	21 (7.9)	32 (12.0)	15 (5.6)	11 (4.1)	2 (0.8)	2 (0.8)
**Vancomycin**	29 (10.9)	25 (9.4)	10 (3.8)	7 (2.6)	2 (0.8)	0 (0)
**Metronidazole**	2 (0.8)	0 (0)	10 (3.8)	2 (0.8)	0 (0)	0 (0)
**Gentamycin**	1 (0.4)	**130 (48.9)**	1 (0.4)	0 (0)	0 (0)	0 (0)
**Colistimethate**	0 (0)	0 (0)	0 (0)	1 (0.4)	0 (0)	0 (0)
**Piperacillin–Tazobactam**	1 (0.4)	0 (0)	0 (0)	2 (0.8)	0 (0)	0 (0)
**Cefotaxime**	3 (1.1)	16 (6.0)	4 (1.5)	1 (0.4)	0 (0)	0 (0)
**Cilastatin/imipenem**	9 (3.4)	0 (0)	7 (2.6)	1 (0.4)	0 (0)	0 (0)

**Table 5:** The group and name of antibiotics changed.

A. Group of antibiotics changed (*n* = 120).				
**Antibiotic Group**	**Changed 1** ***n* (%)**	**Changed 2** ***n* (%)**	**Changed 3** ***n* (%)**	**Changed 4** ***n* (%)**
**Penicillin**	8 (6.7)	2 (1.7)	1 (0.8)	0 (0)
**Penicillin–beta lactamase**	1 (0.8)	1 (0.8)	0 (0)	0 (0)
**Glycopeptide**	41 (34.2)	16 (13.3)	5 (4.2)	0 (0)
**Dehydropeptidase/Carbapenem**	**17 (14.2)**	**39 (32.5)**	**11 (9.2)**	**4 (3.3)**
**Aminoglycosides**	34 (28.3)	18 (15.0)	**4 (3.3)**	0 (0)
**3rd-generation Cephalosporin**	11 (9.2)	6 (5.0)	2 (1.7)	2 (1.7)
**Nitroimidazole**	2 (1.7)	1 (0.8)	2 (1.7)	1 (0.8)
**Polymyxins**	**4 (3.3)**	0 (0)	1 (0.8)	0 (0)
**Macrolide**	1 (0.8)	0 (0)	0 (0)	0 (0)
**Sulfonamide/diaminopyridine**	1 (0.8)	0 (0)	3 (2.5)	1 (0.8)
B. Name of antibiotics changed (*n* = 120).				
**Antibiotic**	**Changed #1** ***n* (%)**	**Changed #2** ***n* (%)**	**Changed #3** ***n* (%)**	**Changed #4** ***n* (%)**
**Ampicillin**	8 (6.7)	2 (1.7)	1 (0.8)	0 (0)
**Amikacin**	26 (21.7)	9 (7.5)	3 (2.5)	0 (0)
**Meropenem**	8 (6.7)	34 (28.3)	8 (6.7)	**4 (3.3)**
**Vancomycin**	41 (34.2)	16 (13.3)	5 (4.2)	0 (0)
**Metronidazole**	2 (1.7)	1 (0.8)	2 (1.7)	1 (0.8)
**Gentamycin**	8 (6.7)	9 (7.5)	1 (0.8)	0 (0)
**Colistimethate**	**4 (3.3)**	0 (0)	1 (0.8)	0 (0)
**Piperacillin–Tazobactam**	1 (0.8)	1 (0.8)	0 (0)	0 (0)
**Ceftriaxone**	2 (1.7)	0 (0)	1 (0.8)	0 (0)
**Cefotaxime**	9 (7.5)	6 (5.0)	1 (0.8)	2 (1.7)
**SMX/TMP**	1 (0.8)	0 (0)	3 (2.5)	1 (0.8)
**Azithromycin**	1 (0.8)	0 (0)	0 (0)	0 (0)
**Cilastatin/imipenem**	9 (7.5)	5 (4.2)	3 (2.5)	0 (0)

**Table 6:** Resistance patterns to antibiotics.

Antibiotic Resistance to Pathogens	*n* (%)	*E-coli*	*Enterobacter*	*K. pneumonia*	*Micrococcus*	*MRSA*	*Staph. Epidermis*	*Viridans streptococci*	*Staphylococcus hominis*	*Acinetobacter baumannii*	*Sphingomonas*	*Klebsiella oxytoca*	*Streptococcus pneumoniae*	*Streptococcus mitis*	*Staphylococcus haemolyticus*	*Enterococcus*	*Coagulase-negative Staphylococci*	*Staphylococcus aureus*
		*n*																
**Ampicillin**	**15 (5.6)**	**2**		**1**		**3**	**5**	**3**	**1**									
**Gentamicin**	**22 (8.3)**	**1**		**7**		**1**	**1**	**2**	**2**	**1**	**1**	**1**	**2**			**1**	**2**	
**Penicillin**	**41 (15.4)**				**11**		**7**	**11**	**3**				**1**	**1**	**4**		**3**	
**Imipenem**	**16 (6.0)**			**12**						**3**	**1**							
**Meropenem**	**14 (5.3)**			**10**						**2**	**1**	**1**						
**Ceftriaxone**	**16 (6.0)**	**1**		**1**		**1**	**5**	**3**				**1**	**2**	**1**	**1**			
**Cefotaxime**	**37 (13.9)**	**4**		**11**		**1**	**5**	**9**		**3**	**1**		**1**	**1**	**1**			
**Ceftazidime**	**27 (10.2)**	**3**		**11**		**1**	**5**	**3**		**1**	**1**	**1**			**1**			
**Cefepime**	**25 (9.4)**	**2**		**11**		**1**	**5**	**2**		**1**	**1**	**1**			**1**			
**Cefoxitin**	**35 (13.2)**	**1**		**12**			**7**	**5**	**3**						**4**		**3**	
**Erythromycin**	**20 (7.5)**			**5**			**4**	**3**	**1**				**1**		**4**	**1**		**1**
**Oxacillin**	**33 (12.4)**			**11**			**7**	**4**	**3**				**1**		**4**		**3**	

## Data Availability

The data underlying this article will be shared upon reasonable request to the corresponding author.
